# Development and application of a field knowledge graph and search engine for pavement engineering

**DOI:** 10.1038/s41598-022-11604-y

**Published:** 2022-05-12

**Authors:** Zhihao Yang, Yingxin Bi, Linbing Wang, Dongwei Cao, Rongxu Li, Qianqian Li

**Affiliations:** 1 National Center for Materials Service Safety, University of Science and Technology Beijing (USTB), Beijing, 100083 China; 2 Research Institute of Highway Ministry of Transport, Beijing, 100088 China; 3 School of Economics and Management, University of Science and Technology Beijing (USTB), Beijing, 100083 China; 4 Joint USTB Virginia Tech Lab on Multifunctional Materials, Department Civil and Environmental Engineering, USTB, VA Tech, Blacksburg, VA 24061 USA; 5 School of Materials Science and Engineering, Chang’an University, Xi’an, 710018 China

**Keywords:** Engineering, Civil engineering

## Abstract

Integrated, timely data about pavement structures, materials and performance information are crucial for the continuous improvement and optimization of pavement design by the engineering research community. However, at present, pavement structures, materials and performance information in China are relatively isolated and cannot be integrated and managed. This results in a waste of a large amount of effective information. One of the significant development trends of pavement engineering is to collect, analyze, and manage the knowledge assets of pavement information to realize intelligent decision-making. To address these challenges, a knowledge graph (KG) is adopted, which is a novel and effective knowledge management technology and provides an ideal technical method to realize the integration of information in pavement engineering. First, a neural network model is used based on the principle of deep learning to obtain knowledge. On this basis, the relationship between knowledge is built from siloed databases, data in textual format and networks, and the knowledge base. Second, KG-Pavement is presented, which is a flexible framework that can integrate and ingest heterogeneous pavement engineering data to generate knowledge graphs. Furthermore, the index and unique constraints on attributes for knowledge entities are proposed in KG-Pavement, which can improve the efficiency of internal retrieval in the system. Finally, a pavement information search engine based on a knowledge graph is constructed to realize information interaction and target information matching between a webpage server and graph database. This is the first successful application of knowledge graphs in pavement engineering. This will greatly promote knowledge integration and intelligent decision-making in the domain of pavement engineering.

## Introduction

In 1984, the U.S. government included the long-term pavement performance (LTPP) research project in the long-term highway research program, and implemented the Strategic Highway Research Program (SHRP) three years later^1,2^. The purpose of the SHRP is to provide better pavement technology in the 21st century, and making pavements safer and more durable^3^. Furthermore, in 1989, the LTPP database began to collect information on pavement structures, materials and service-performance mainly in North America^4^. At present, the LTPP database provides an important data support for pavement structures and materials design, service-performances prediction and pavement maintenance decision-making in North America^5^.

In January of 2022, the total mileage of highways in China has exceeded 160,000 km, and the total mileage of pavements in operation has exceeded 5 million km^6^. And the total mileage of highways ranked first in the world. However, the most serious problem is that the integrated pavement information management and search system of highway industry, which is similar to the LTPP database, has not been established for the information of pavement structures, materials, construction process, performance monitoring results and maintenance measures. On the one hand, a great deal of valid data is wasted. The pavement designers and researchers cannot show the intrinsic relationship between pavement structures, materials and performance monitoring results when making pavement scheme decisions. It also limits the possibility of further optimizing pavement structures and materials design schemes. On the other hand, the non-sharing of information has further restricted the application of artificial intelligence, precise search, assistant decision support technology in the field of pavement engineering. That is to limit the intelligent development of the pavement industry.

Because there are various sources and contents of pavement information, it brings a great challenge to the interconnection of pavement information under the background of big data. Therefore, according to the principle of knowledge organization under the background of big data, it is necessary to explore the method of pavement information interconnection which could meet the development and change of network information resources and the cognitive needs of users from a new perspective^7^. Furthermore, it also should reveal the integrity and relevance of user cognition from a deeper level.

In 2012, Google proposed the concept of knowledge graph (KG), which is used to enhance the performance of the database and improve the retrieval ability^8^. At the same time, Google has also released a high-performance graph database, which is Neo4java or Neo4j. Knowledge graph is an important research field of artificial intelligence^9^. It can realize the interconnection of knowledge with its superior semantic processing and open-interconnection ability. Knowledge graph is a modern theory that combines the theories of applied mathematics, graphics, information visualization technology, information science and other disciplines with the methods of bibliometric citation analysis and co-occurrence analysis, and also uses visual mapping to vividly display the core structure, development history, frontier areas and overall knowledge structure of the discipline to achieve the purpose of multidisciplinary integration^10–12^. Knowledge -graph describes entities, attributes and their relationships in the objective world in a relatively fixed and structured form, which can express knowledge or information in a form closer to the human cognitive world^13^. It also provides an ability to better organize, manage and understand large amounts of knowledge and information. In other words, knowledge graph is a kind of knowledge representation which could formalize the description of knowledge entities and the relationship between them^14,15^. And its essence is semantic web rather than semantic network^16^. Semantic network is only a way to express the knowledge, that is, to store and display knowledge in the form of graph, which cannot realize the reasoning of knowledge^17^. While the goal of the semantic web is to realize the interconnection of knowledge and semantics, which can realize the expression of critical knowledge and deep knowledge^18–20^. That is, it can not only identify words and concepts, but also identify the logical relationship between them, which can make the data interaction more efficient and valuable. Therefore, based on the characteristics of knowledge graph, the functions of intelligent search, reasoning and intelligent question answering of knowledge can be realized. Knowledge graph, together with big data and deep learning, has become one of the core drivers for the development of the Internet and artificial intelligence.

According to the type of knowledge, knowledge graphs can be divided into the general knowledge graphs and the professional domain knowledge graphs. The general knowledge graphs cover a wide range of knowledge, such as Baidu Encyclopedia, Freebase, Wordnet, Babelnet, etc. However, the professional domain knowledge graphs pay more attention to the types of professional knowledge involved, such as IMDB, MusicBrainz, TMC-knowledge graph^21^, KG-COVID-19^22^, etc. The application of knowledge graphs show that knowledge graphs can provide knowledge visualization, knowledge retrieval, knowledge recommendation and other knowledge services, which is conducive to sharing, interpretation and utilization of knowledge.

**Figure 1:**
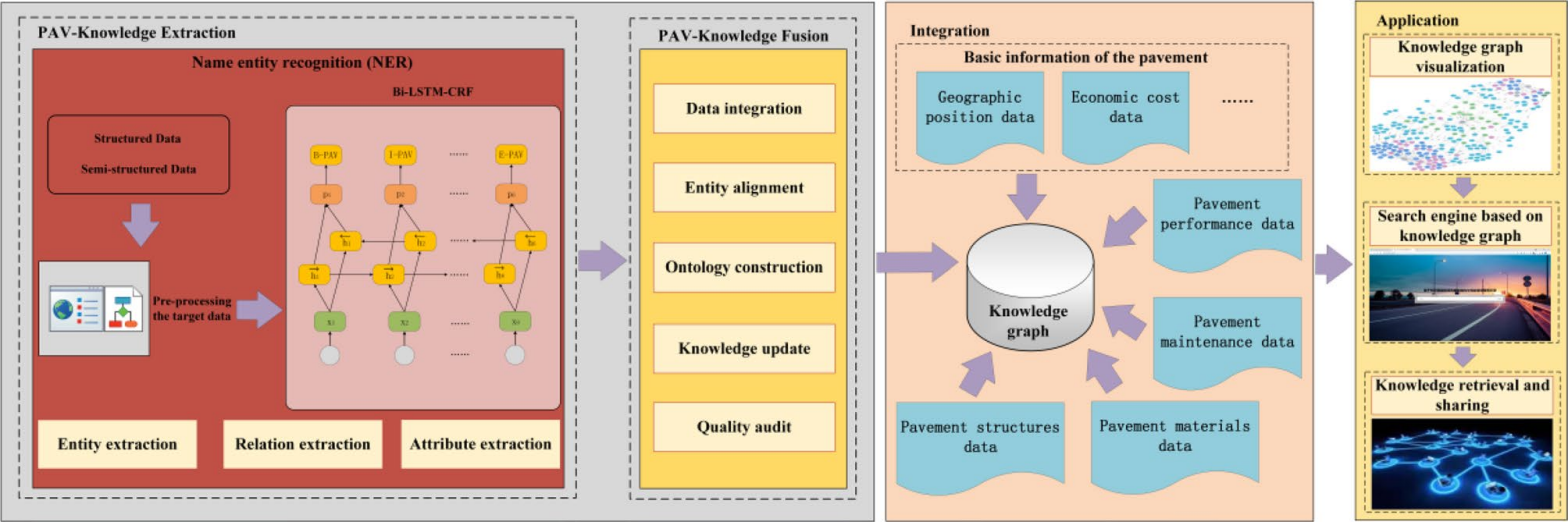
The technical route of the article.

To tackle the formidable challenge of bringing together disparate sources of pavement information and to collect useful knowledge from them, a knowledge graph was used in the article. The Neo4j graph database was also used to store and visualize the pavement information extracted by the knowledge graph. However, the pavement information in the Neo4j graph database needs to be searched through its specific system, which greatly limits the popularization and use of this database. Therefore, a webpage search engine system based on the Neo4j graph database was built. To update the information in the graph database without restriction and to fully exploit the pavement information, the graph database and search engine are both open access systems. The research of this paper can not only realize the centralized management and open access of pavement information in China but also provide information support for the research and decision-making activities of researchers and designers. More importantly, this study also fills the gap in the application of knowledge graphs in pavement engineering. The technical route of this study is shown in Fig. 1.

As can be seen from Fig. 1, this technical route mainly includes three steps. The first step is the extraction and fusion of knowledge. The second step is to construct the knowledge dataset. The third step is the construction and application of the knowledge graph and search engine. And through these three steps, this paper will construct a shared management platform of pavement information, and realize the integration and management of pavement information.

## Constructing the knowledge dataset

Before constructing KG-Pavement, it is necessary to construct a knowledge dataset. The workflow can be divided into four steps: fetching the target data, preprocessing the target data, extracting the knowledge, and fusing the knowledge.

### Fetching the target data

Before constructing a knowledge graph, it is necessary to fetch effective information from complicated text data, which is called knowledge discovery^23^. This is also a key step in constructing a knowledge dataset. The information on pavement structures, materials and performances can be fetched from networks, text, the literature and so on. In this paper, knowledge was mainly fetched from pavement inspection report text provided by the Research Institute of Highway Ministry of Transport and National Center for Quality Supervision and Inspection of Roads and Bridges. For the network data, Scrapy, the crawler framework in Python, was used to fetch pavement information. The specific method selected information resources from webpages. Then, the uniform resource locator (URL) of the information resource was imported into the queue of the URL. Finally, the data links in the queue were parsed in turn, and the effective data were extracted into the dataset. In addition, a focused crawler was used to avoid fetching invalid data. This is because focused crawlers can fetch data from subject-specific content and specific-industry webpages, while content from other industries is not considered. That is, target data are fetched only for the specific topic of pavement structures, materials and performance monitoring information, while other types of data are not considered.

### Preprocessing the target data

After fetching the target data, it is necessary to standardize the expression of the target data according to the design requirements of the knowledge graph to improve the efficiency of the knowledge extraction process. This makes the target data perfect, accurate and effective. Data preprocessing includes word segmentation and part-of-speech standards, which are the basis of named-entity recognition and relation extraction. Data preprocessing uses spaCy, which is an open-source library for advanced natural language processing (NLP) in Python. It contains neural network algorithm models that can be used for data text language processing, such as word segmentation, tagging, parsing, text classification and named-entity recognition.

### Extracting knowledge

**Figure 2:**
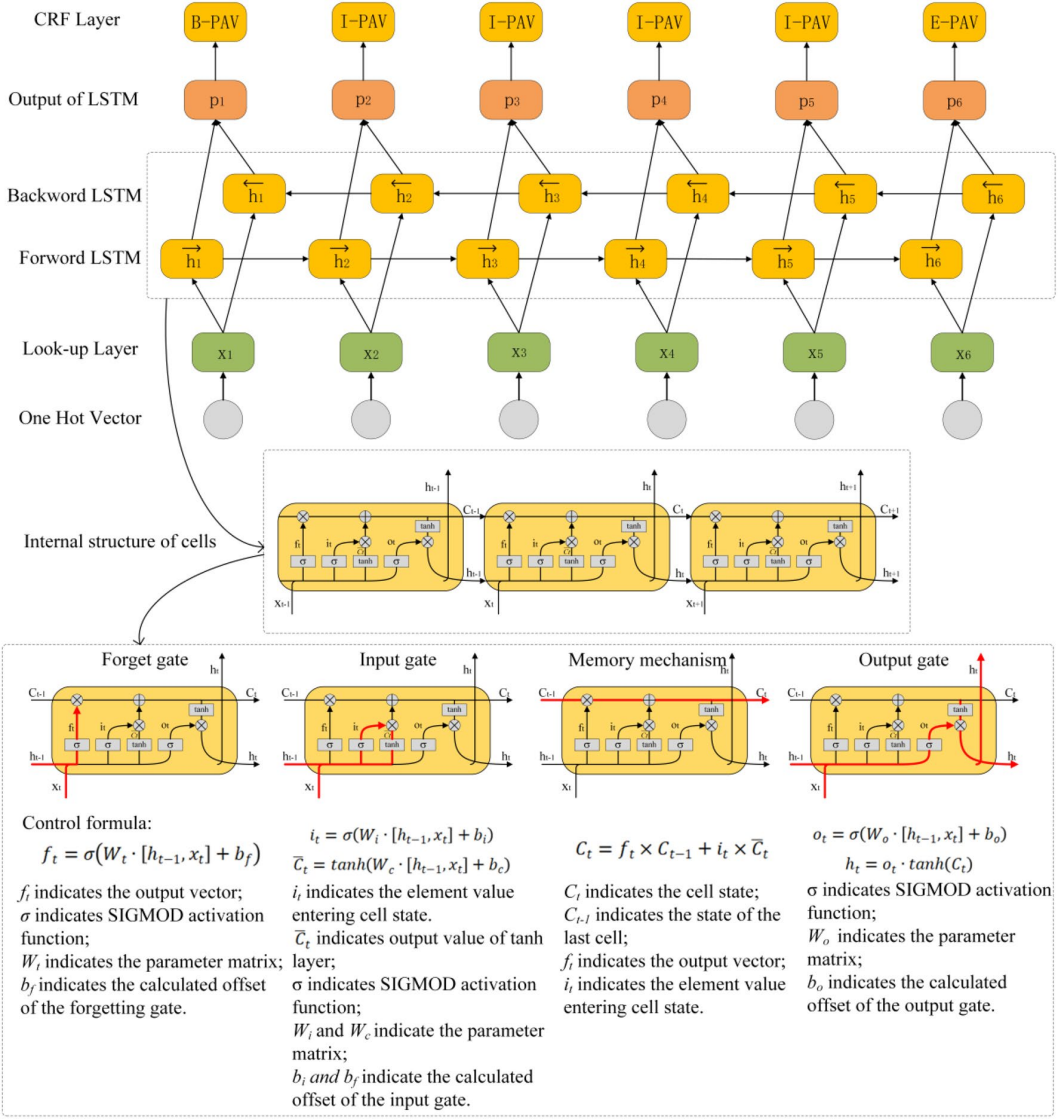
The Bi-LSTM CRF model and structure of the cells in LSTM.

Named-entity recognition and relationship extraction are key steps in the process of knowledge extraction^24^. Their purpose is to identify the boundaries of types of knowledge entities with specific meanings in the text^25^, such as names, dates, places, and organizations and the relationships between them, and assign corresponding predefined category labels for classification and statistics. At present, the deep learning method is the most commonly used knowledge extraction method. This method transforms knowledge extraction into a preset sequence calibration process. That is, to identify and classify different knowledge entities and relationships, the rules of the preset sequence calibration are assigned to different entities and relationships in the form of category labels^26^. Among them, the Bi-LSTM-CRF model proposed by Huang et al. is the most famous knowledge extraction model^27^. It is the first model to use a deep learning method for named-entity recognition and relationship extraction, which makes the process of knowledge extraction more efficient and reduces the dependence on word vectors. The Bi-LSTM-CRF model is shown in Fig. 2.

As shown in Fig. 2, the Bi-LSTM-CRF model was obtained by combining the forward-cyclic long-short term memory (LSTM) model and the reverse-cyclic LSTM model with the conditional random field (CRF) model. Among them, the LSTM model is composed of multiple neuron cells, and its core is the gate mechanism and memory mechanism. In other words, each neuron cell is jointly controlled by a forget gate, an input gate, a memory mechanism and an output gate to control the input value, output value and information transmission^28^. The memory mechanism can minimize the attenuation of information. Meanwhile, through the gate mechanism, neuron cells can filter out unimportant information and update important information. Moreover, this filtering and updating have little effect on the cell state. Therefore, it can solve the problem of long-term dependency and gradient disappearance between text.

The asphalt pavement structure and performance information after knowledge extraction should be structured data. That is, the data information can be unified at the triple level of the resource description framework (RDF).

### Knowledge fusion

Due to the different sources of knowledge, there may be redundant and repetitive knowledge, a problem that must be solved by knowledge fusion. The focus of knowledge fusion is to achieve entity alignment. For the different sources of knowledge, the triples of the entity can be integrated into the knowledge graph to be expanded after the process of entity alignment to expand the scale of the knowledge graph. The process of entity alignment includes a knowledge representation learning model, alignment model and type matching constraints. First, the PTransDW model was mapped to the vector space by using the relationship and expressed as a real-valued vector. Then, the similarity was calculated after it was embedded into the same semantic space. Finally, the types of entities were constrained for knowledge integration.

## Constructing the knowledge graph and search engine

A knowledge graph can show the process of knowledge development and the relationship between the structure through a series of different graphics and can also describe knowledge resources and their carriers by visualization technology. Its nodes represent entities or concepts, and its edges represent various semantic relationships between entities or concepts. This can accurately capture, analyze and mine relationship attributes such as pavement structure information, material information and performance information. Therefore, the knowledge graph of a pavement structure and performance information is a mapping network in a big data environment^29,30^.

### Graph database

To construct pavement information knowledge graphs, it is necessary to use appropriate methods to store the extracted data. A graph database is a data storage system based on Euler theory and spectrogram theory. Euler theory is a theory about the congruence property and describes the unique laws among the vertex number, face number and edge number in a polyhedron. Spectrogram theory mainly studies the topological properties of graphs based on the properties of adjacency, Laplacian, signless Laplacian and correlation matrices. The data in complex networks can be accurately mined through Euler theory and spectrogram theory to establish correlations between the parameters of the graphs.

**Figure 3:**
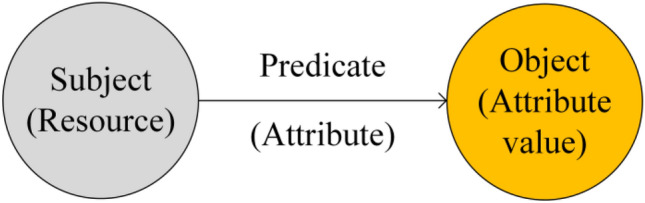
The three-tuple.

A graph database adopts the data storage mode based on a resource description framework (RDF). ARDF describes knowledge in the form of a three-tuple. The structure of three-tuple is shown in Fig. 3.

Figure 3 shows that the three-tuple can be expressed as “subject—predicate—object” or “resource—attribute—attribute value”. That is, the knowledge can be decomposed and stored in the form of “subject—predicate—object” or “resource—attribute—attribute value”. In addition, the three-tuple can also describe knowledge as a set, that is, $\mathrm{G}=\left(\mathrm{E},\mathrm{R},\mathrm{S}\right)$. $\mathrm{E}$ denotes a set of knowledge entities. $\mathrm{E}=\left\{{\mathrm{e}}_{1},{\mathrm{e}}_{2},\cdots {\mathrm{e}}_{\mathrm{n}}\right\}$ indicates that the set contains a total of $\mathrm{n}$ knowledge entities. $\mathrm{R}$ denotes a set of relationships between entities. $\mathrm{R}=\left\{{\mathrm{r}}_{1},{\mathrm{r}}_{2},\cdots {\mathrm{r}}_{\mathrm{m}}\right\}$ indicates that the set contains a total of $\mathrm{m}$ entity relationships. $\mathrm{S}$ denotes a set of the three-tuples, that is, $\mathrm{S}\subseteq \mathrm{E}\times \mathrm{R}$.

This data storage model can represent the stored information in the form of a graphic structure after visualization. That is, compared with a relational database, a graph database can directly express the relationship between entities through nodes and edges. The nodes and edges can contain multiple attributes, so the attribute characteristics of entities and relationships can also be expressed intuitively. With accelerating globalization and information relevance, graph databases have received increasing attention from researchers in the database field because of their data relevance and convenience in dealing with complex dynamic data, and their market share is also increasing. There are many well-known graph databases, such as Neo4j, OrientDB, Dgraph, and ArangoDB. Among them, Neo4j is the most representative graph database and has occupied first place in the global graph database market for many years^31^ (see Table 1).

**Table 1:** The popularity ranking of graph database database management systems in March 2022.

Rank			Database	Score		
Mar. 2022	Feb. 2022	Mar. 2021		Mar. 2022	Feb. 2022	Mar. 2021
1	1	1	Neo4j	59.67	+ 1.43	+ 7.35
2	2	2	Microsoft Azure Cosmos DB	40.90	+ 0.94	+ 8.49
3	3	3	ArangoDB	5.61	+ 0.21	+ 0.55
4	4	5	Virtuoso	5.57	+ 0.18	+ 2.70
5	5	4	OrientDB	4.92	−0.10	+ 0.22
6	7	7	GraphDB	2.84	−0.09	+ 0.57
7	6	8	Amazon Neptune	2.69	−0.30	+ 0.83
8	8	6	JanusGraph	2.47	+ 0.11	+ 0.04
9	9	11	TigerGraph	2.18	−0.06	+ 0.68
10	10	10	Stardog	1.90	−0.08	+ 0.39

Compared with other graphic databases, the main advantage of Neo4j is that it focuses on solving the performance degradation problem of traditional database with a large number of connections when querying. It traverses nodes and edges at the same speed, and its traverse speed has nothing to do with the amount of data that constitutes the graph. Therefore, Neo4j (version 4.1.4) was used as the graph database for data storage in this article.

### Design principles

To reasonably organize knowledge, it is necessary to define the pattern of knowledge mapping to better describe the knowledge itself and the relationship between the knowledge. This process is called knowledge modelling. Knowledge modelling is an important process used to establish the conceptual model of a knowledge graph. In general, the same knowledge can have a variety of knowledge modelling methods. An efficient knowledge modelling method can reduce redundant knowledge and improve the utilization efficiency of the knowledge graph. Therefore, it is necessary to combine the characteristics of knowledge and applications to carry out knowledge modelling.

There are usually two methods used to carry out knowledge modelling. The first method is a top-down knowledge modelling method. First, the knowledge model is defined from the top concept to construct the knowledge graph. Then, it is gradually refined downwards to form a well-structured classification level. Finally, the entity is matched with the concept. This method is usually used to construct field knowledge graphs for specific industries. That is, the knowledge model is defined according to the characteristics of the industry. Another method is a bottom-up knowledge modelling method. The first step is to generalize organizational knowledge entities to form underlying concepts. Then, the underlying concepts are gradually clustered to form the upper concepts. This method relies on an open-connection dataset to automatically learn from this structured knowledge. This method is usually used to construct a general knowledge graph. Therefore, the top-down method was adopted for knowledge modelling.

**Figure 4:**
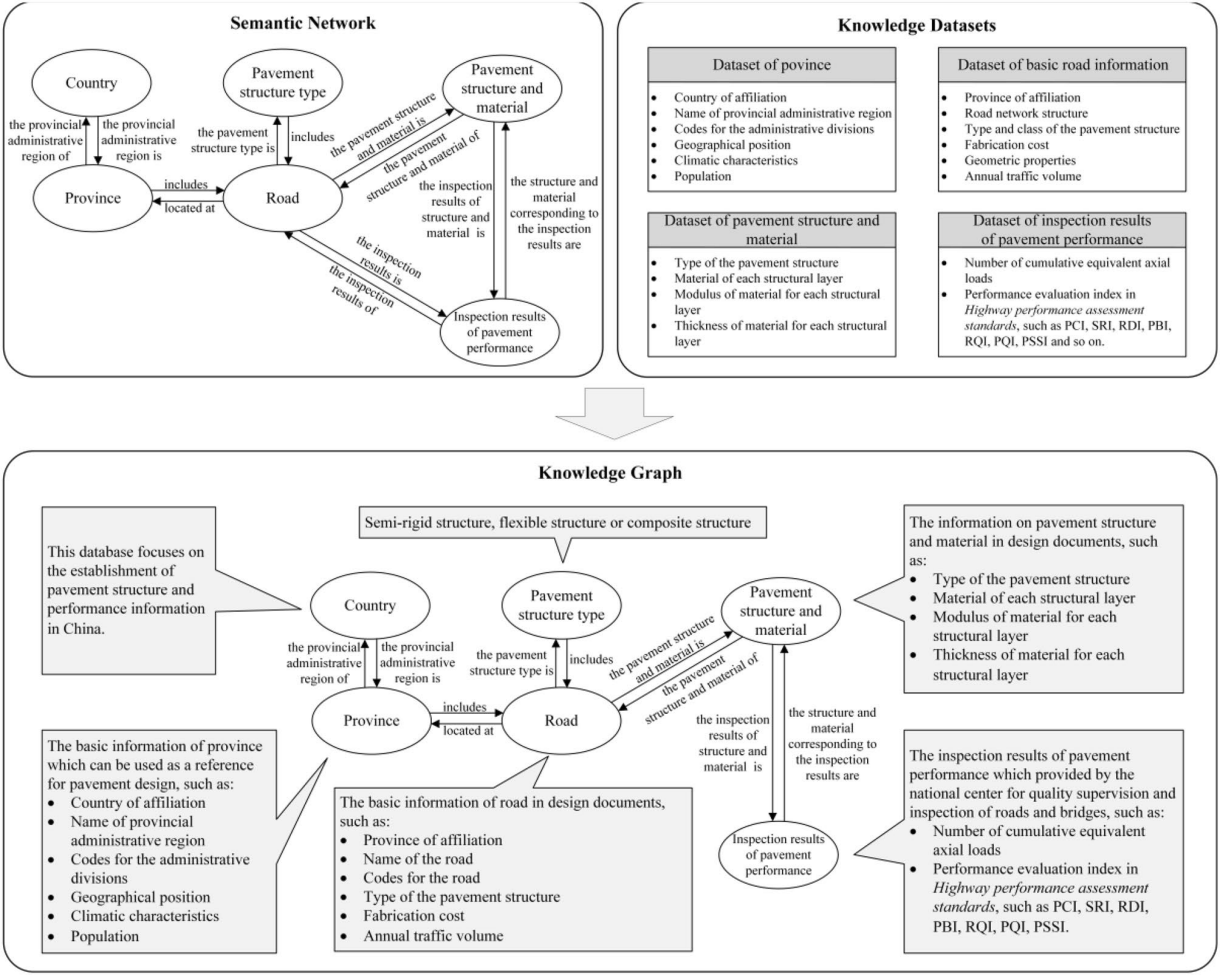
Construction of knowledge graph for pavement information.

Meanwhile, the purpose of this research is to construct a knowledge graph of the pavement structure and performance information based on the semantic network and the knowledge datasets, that is, KG-Pavement. The knowledge graph was designed to support data updates, interoperability, and the ability to flexibly match and query data. The workflow is shown in Fig. 4.

Figure 4 shows that the workflow can be summarized as follows: the existing knowledge datasets are integrated to form a knowledge graph of the pavement structure and performance information that uses a semantic network as the skeleton to realize knowledge storage, knowledge association, knowledge reasoning and intelligent knowledge services. Among them, the most important is the construction of the dataset. As shown in Fig. 4, four datasets are established in this paper. The main objective of the province dataset is to collect the basic information on the administrative area where the pavement is located. The province dataset includes six pieces of information, such as climate characteristics, local specifications, population, economic scale and so on. According to different types and levels, the dataset of basic road information includes at least 8 pieces of information, such as province of affiliation, name of the road, road network structure, type and class of the pavement structure, fabrication cost, geometric properties, annual traffic volume, length of pavement and so on. Datasets of pavement structures and materials are one of the most important datasets. They contain four basic pieces of information, such as the type of pavement structure, material of each structural layer, modulus of the material and poisson ratio for each structural layer, and thickness of each structural layer. Another of the most important datasets is the dataset of the inspection results of pavement performance. It mainly contains two aspects of data, namely, the number of cumulative equivalent axial loads and the performance evaluation index. Meanwhile, the original calculation data of each evaluation index were also included in the corresponding dataset of the pavement performance, such as rutting depth, deflection value, international flatness index, etc. It should be noted that the inspect results of pavement performance have corresponding mileage labels. In other words, when the pavement is divided into different inspection sample units for save effort and time, the inspect results of different sample units are also different for a same pavement section. And the inspection results of these sample units are all stored in the dataset according to the corresponding mileage labels.

Before constructing the knowledge graph, it is necessary to define the attributes of entities and the relationship between entities. First, based on the knowledge base, a five-layer node relationship model is designed. Then, the pavement structure and material information are matched with the performance information, and the pavement structure and material information are matched with the basic road information. Finally, the pavement structure and performance information are clustered according to the administrative region, type and class of pavement structure.

In addition, considering that different sections of the same road may have different structures, the relationship of the pavement performance information node with the structure information node is built rather than with the road information node. The arrow indicates the relationship between nodes. That is, the five-layer node relationship is defined. At the same time, to facilitate the establishment of the relationship between nodes, the codes for the roads are defined according to the codes for the administrative divisions. For example, the code for the administrative divisions of Shandong Province is "37", and the code for the Jinan-Qingdao Expressway is defined as "37-001". It is important to note that each node or relationship generated in Neo4j has a system-assigned ID that starts from 0 and is globally unique. The code for the road created is only an attribute value of a node or relationship and does not conflict with or affect others with the system-assigned ID.

### Methods of construction and optimization

The Cypher programming language was used to import the integrated data in the Neo4j program to construct the knowledge graph. The main coding examples for constructing the knowledge graph are shown in Table 2. This method is simple and convenient, but a large amount of data needs to be processed and integrated in advance. It is suitable for constructing a large-scale knowledge graph.

**Table 2:**
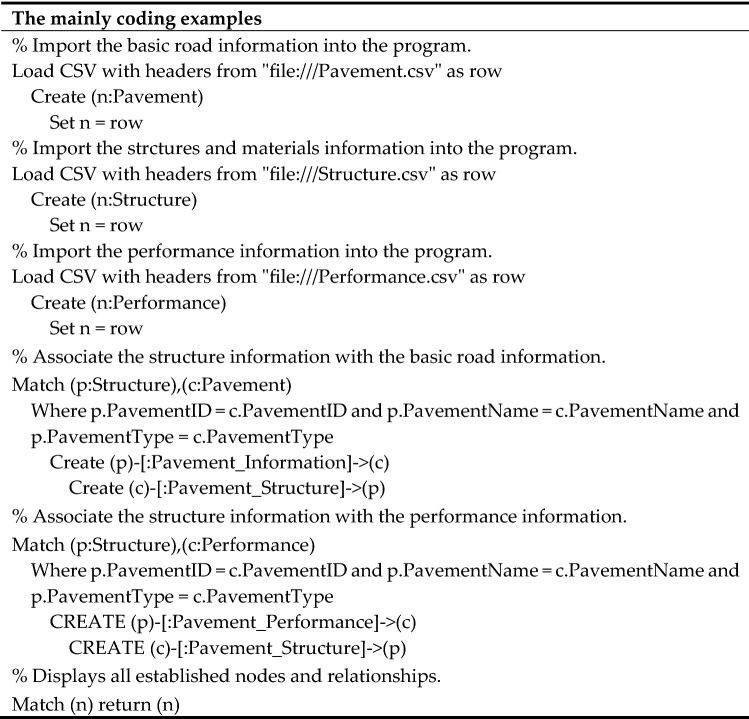
The mainly coding examples for constructing knowledge graph.

Information on 106 pavements was included in the knowledge graph. Each pavement included basic information nodes, structural and material nodes, and performance nodes, and a bidirectional correlation was established between these nodes. Meanwhile, the pavement information was clustered and correlated according to the type and functional class of the structure and the administrative region. Furthermore, in a large-scale knowledge graph, the retrieval efficiency of data is usually improved by constructing internal indices and setting unique constraints on attributes.

According to a large number of experimental results, the retrieval efficiency can be improved by 30% ~ 300% by constructing internal indices. However, the establishment of indices can also cause a decrease in the data update rate. Therefore, according to the query requirements, the fields with more queries should be indexed. Multiple indices can be established with the same label.

Furthermore, the constraint and assertion syntax in the Cypher programming language are used to establish unique constraints on attributes for important labels. In this process, the system will automatically create indices. That is, the procedure is equivalent to creating an index with unique constraint properties for the label. The main coding examples are shown in Table 3.

**Table 3:** The mainly coding examples for the methods of optimization.

Projects	The mainly coding examples
Create an index labeled as Pavement	Create index on:Pavement ( )
Use index to query target data	Match (n:Pavement) Using Index n:Pavement Where n.PavementName = ' '
Create attribute unique constraints for the label which is PavementName	Constraint on Create constraint on (p: Pavement) Assert p.PavementName is unique
Delete the index	Drop index on:Pavement ( )
Delete the constraint	Drop constraint on ( ) assert p.PavementName is unique

### Visualization of the knowledge graph

**Figure 5:**
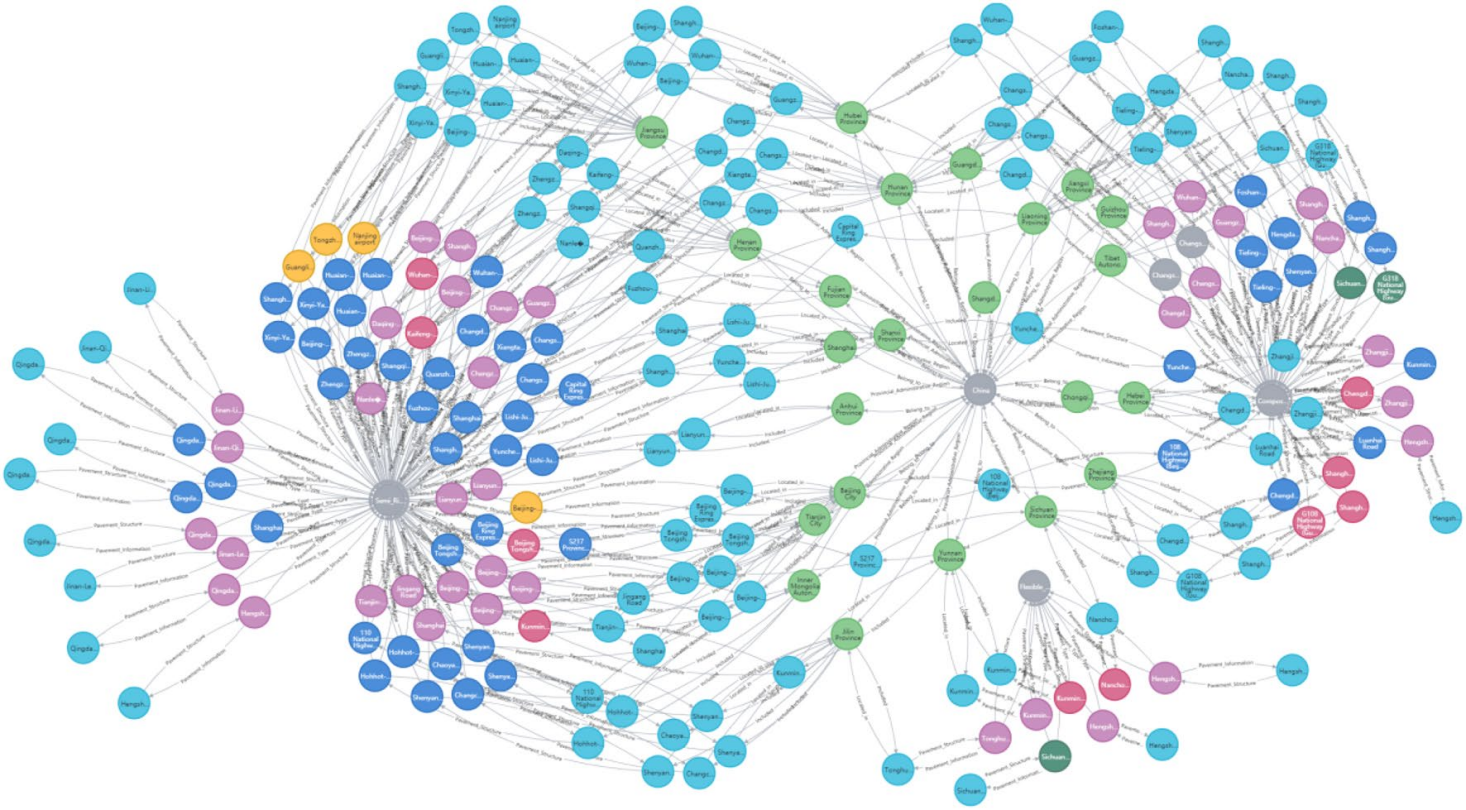
The knowledge graph of pavement information.

The visualization of the knowledge graph is shown in Fig. 5.

As shown in Fig. 5, through the visualization of the knowledge graph, the attributes of entities and the relationship between the entities can be graphically shown. It can also establish connections between mutual independent knowledge and enhance the connectivity of pavement structures, materials and performance information. This solves the problem of “knowledge islands” formed by the mutual independence of the information for each road in the past. More importantly, the knowledge graph can help users systematize and integrate the existing information and explore the potential relationship between the pavement structure, materials and service performance to better optimize the design and performance prediction of the pavement.

### Constructing the search engine based on the knowledge graph

**Figure 6:**
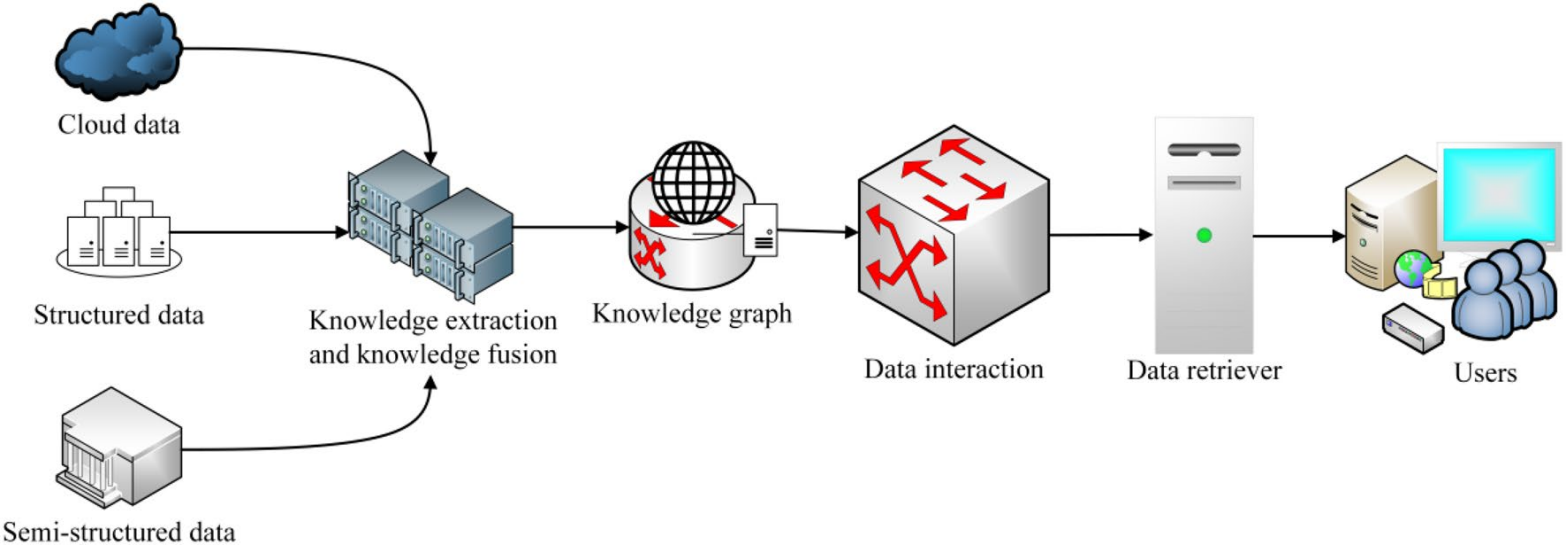
The framework of search engine.

The browser/server mode was used to design the pavement information management and search engine system. The front end of the pavement information management and search engine system uses Vue.js (version 2.6.12) and the Element UI toolkit (version 2.15.6) to construct the webpage interface. The back-end uses Python (version 3.7) and Django (version 2.1.7) to design the program and controls the exchange of information with the Neo4j graph database. Finally, the function of pavement information input, storage and information retrieval can be realized (see Fig. 6).

**Figure 7:**
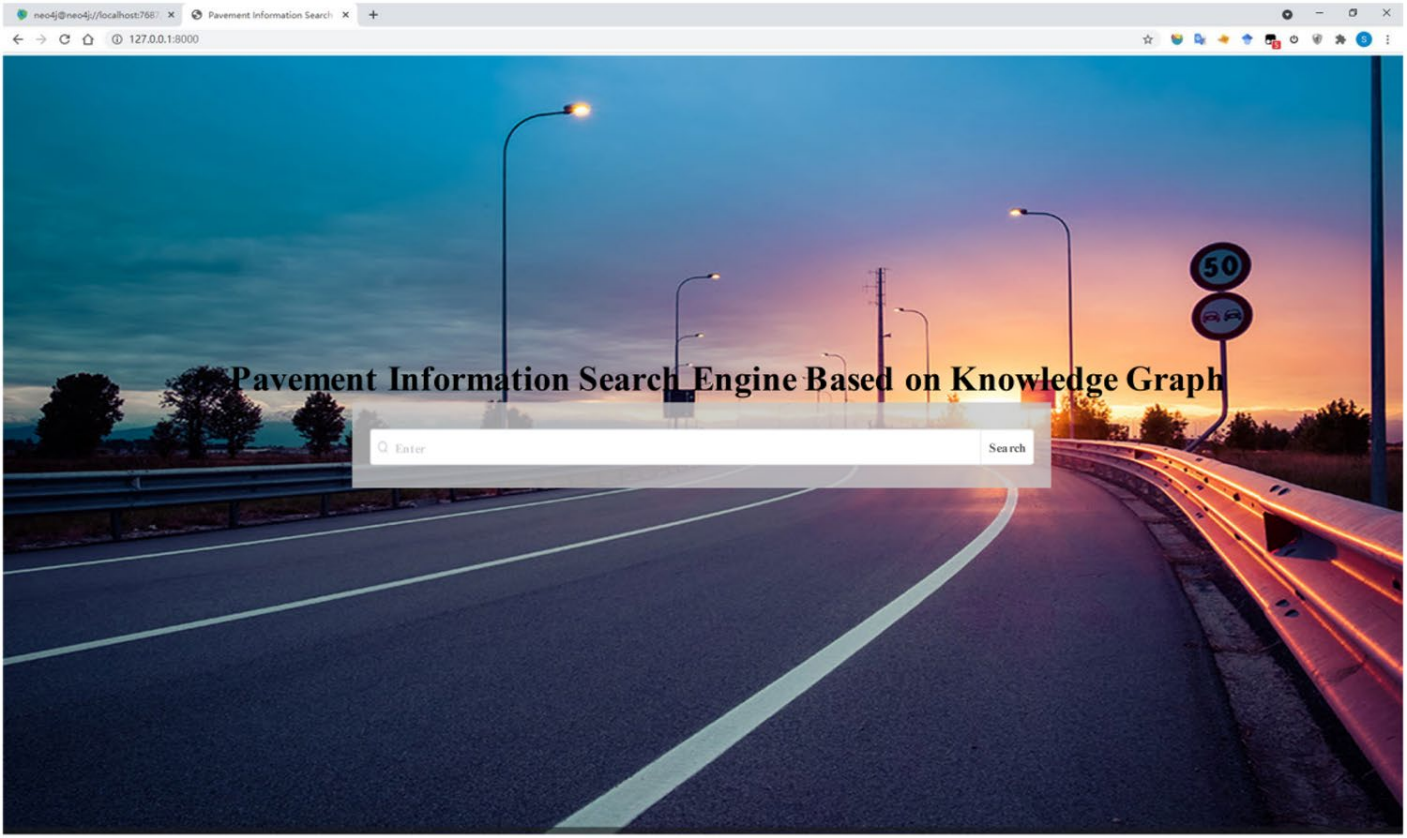
The pavement information search engine based on knowledge graph.

As shown in Fig. 6, users enter the target information in the search engine and pass the target information to the console through the webpage. The console exchanges information with the database and retrieves and matches the target information. Finally, the target information is displayed on the browser page through the server. The home page of the pavement information search engine based on knowledge graph is shown in Fig. 7.

**Figure 8:**
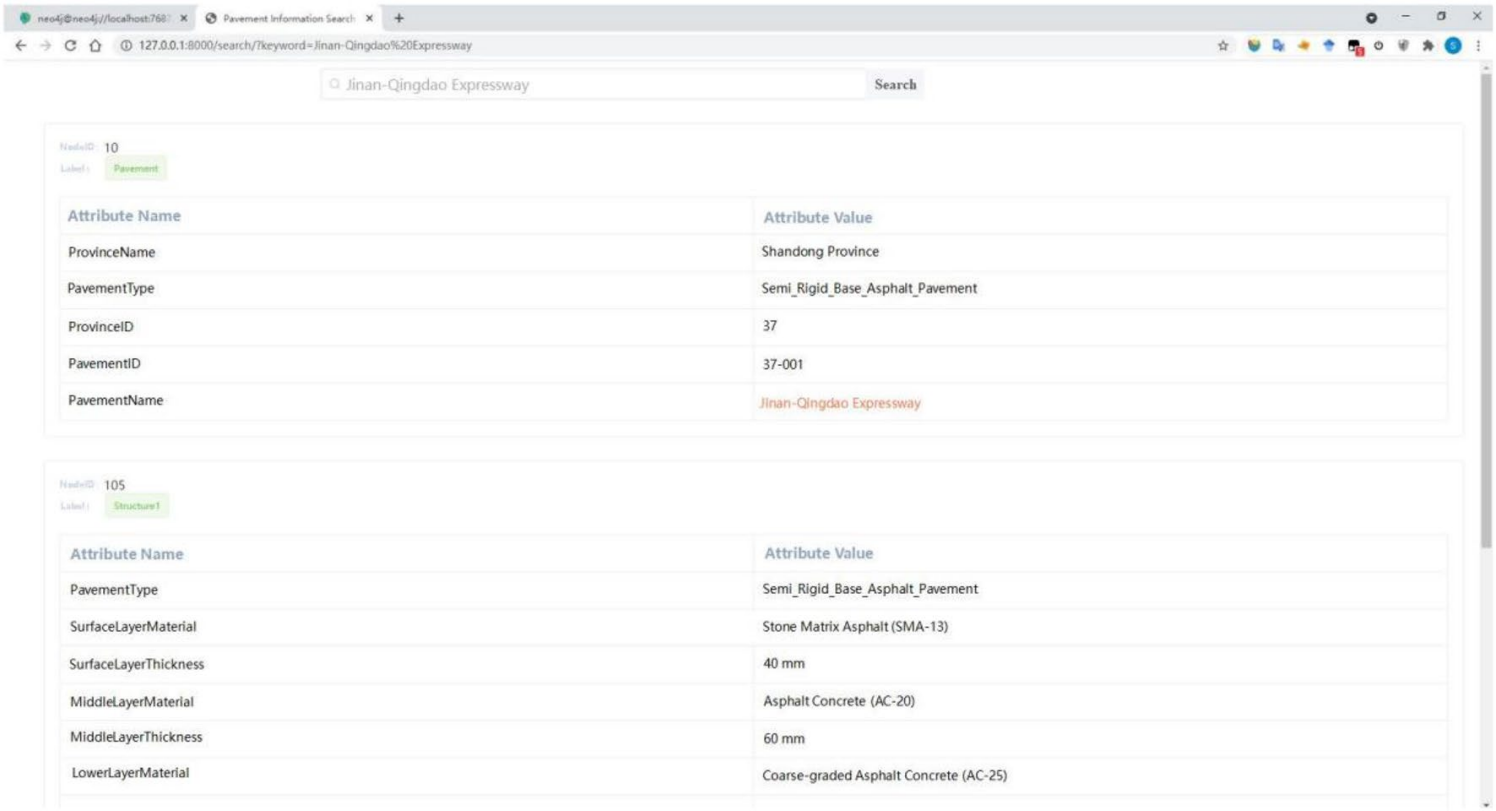
The display of pavement information search results.

The search results of the pavement information search engine based on knowledge graph are shown in Fig. 8.

The information in Fig. 8 shows that the search engine presents the target data well. This shows that the pavement information search engine based on the knowledge graph designed in this paper achieved the design goal.

## Application of KG-Pavement to Analyze a Typical Pavement Structure and the Main Damage Type

According to the classification and analysis of the relationship between the structure and performance of asphalt pavement in the knowledge graph, the relationship between the asphalt pavement structure and the main damage types in China can be summarized (see Table 4).

**Table 4:** The relationship between the asphalt pavement structure and the main damage types.

Structure type	Thickness of asphalt concrete course	The main damage types	The secondary damage type
Semirigid base asphalt pavement	40–60 mm	Fatigue cracking of semirigid base course, reflection crack of semirigid base course	Deformation of subgrade
	60–120 mm	Permanent deformation of asphalt concrete layer, fatigue cracking of semirigid base course, reflection crack of semirigid base course	Reflection crack of semirigid base course
	≥ 120 mm	Fatigue cracking of asphalt concrete course, Permanent deformation of asphalt concrete course	Fatigue cracking of semirigid base course, reflection crack of semirigid base course
Flexible base asphalt pavement	40–60 mm	Permanent deformation of asphalt concrete layer, permanent deformation of base course	Deformation of subgrade
	60–120 mm	Fatigue cracking of asphalt concrete course	Permanent deformation of asphalt concrete layer
	≥ 120 mm	Permanent deformation of asphalt concrete course	Fatigue cracking of asphalt concrete course

The main defects of asphalt pavement can be summarized as the permanent deformation of the asphalt concrete course, fatigue cracking of the semirigid base course, reflection cracking of the semirigid base course and fatigue cracking of the asphalt concrete course.

Through the analysis of inspect results, it can be concluded that the lack of targeted design of pavement structures and materials is the main reason for the frequent occurrence of pavement diseases. For structures, the pavement structures of different sections or regions are mostly similar. And there is no targeted design method of pavement structures for the sections with special traffic volume and long longitudinal slope sections. For materials, modified asphalt mixture is usually used in the upper surface layer, but common asphalt mixture is still used in the middle and lower surface layer. And the middle and lower surface layer are the main areas that bear the shear stress. It leads to a larger rut depth in the middle layer, followed by the lower layer. The rut depth of the upper layer is the smallest. Meanwhile, there is no effective measures to prevent reflection cracks between semi-rigid base and asphalt concrete layer. However, the crack-resistant semi-rigid base are increasingly used in new pavement. In addition, setting a stress absorption course or an oil-rich asphalt thin course between the semi-rigid base and asphalt concrete course can effectively alleviate the adverse effect of reflection cracks in the semi-rigid base on asphalt concrete courses, such as the Er-qin Expressway. It can prolong the service life of pavement. This is an emerging pavement technology in recent years.

Through the knowledge graph, increasing the thickness of the asphalt concrete surface is one of the main characteristics of the development trend of semirigid asphalt pavement in China. Increasing the thickness of the asphalt concrete surface can reduce the extent of damage in the form of a reflection crack in the semirigid base to the pavement structure. However, it will increase the permanent deformation of the asphalt concrete layer. Therefore, anti-rutting asphalt mixtures and high-modulus asphalt mixtures are increasingly used as the materials for the upper and middle surface layers, respectively.

In summary, the clustering and analysis of the data in the KG-Pavement can provide effective data support for pavement design and performance prediction.

## Conclusions

The main results of this research are summarized as follows:

1. Knowledge graphs are a burgeoning technology in artificial intelligence and are rapidly becoming a research hotspot in knowledge management and knowledge reasoning. In this article, knowledge graph technology is proposed in pavement engineering for the first time to realize the management of a large amount of pavement structures, materials and performance information. This article builds a knowledge dataset system and knowledge graph of pavement information. It also presents a technology system of knowledge matching, knowledge acquisition and knowledge services for pavement information. Knowledge graphs are a new technology and foundation for systematic organization, depth analysis based on big data and intelligent management of pavement information knowledge systems. They have broad application prospects in pavement structure design and material performance prediction in pavement engineering.
2. In this paper, a knowledge graph of asphalt pavement information was constructed. The visualization of the knowledge graph was also realized. Furthermore, the index and unique constraints on attributes for knowledge entities in the Neo4j graph database were constructed, which could improve the efficiency of internal retrieval in the system. Finally, a pavement information search engine based on a knowledge graph was presented to realize the information interaction and target information matching between the webpage server and the Neo4j graph database. The research goal of querying pavement structures, materials and performance information through a webpage server is realized.
3. Through the analysis of the pavement information in the knowledge graph, it can be concluded that the main damage forms of asphalt pavement can be categorized as permanent deformation of the asphalt concrete course, fatigue cracking of the semirigid base course, reflection cracking of the surface course and fatigue cracking of the asphalt concrete course. In the past 20 years, the thickness of the asphalt concrete layer of semirigid base asphalt pavement has gradually increased, and the main defects have also changed from reflection cracks in the semirigid base to the permanent deformation of the asphalt concrete layer. This shows that increasing the thickness of the asphalt concrete layer can not only reduce the adverse effect of reflective cracks on pavement performance but also increase the risk of permanent deformation.
4. Analysis of the knowledge graph shows that setting a stress absorption course or oil-rich asphalt thin course between the semirigid base and asphalt concrete course can effectively alleviate the adverse effect of reflection cracks in the semirigid base on asphalt concrete courses, as in the Er-qin Expressway. It can prolong the service life of pavement. However, it will also increase the cost of pavement by 5% ~ 10%. In addition, from the perspective of cost analysis, road construction costs are also increasing.
5. For people working as pavement engineers, KG-Pavement can search for information on the structures, materials, cost, service performance, etc. of active pavement, which can provide data support for pavement structure design. At the same time, it can also facilitate the upload and management of information by researchers to achieve information sharing.

Several aspects presented in this paper warrant further examination: (1) to achieve the universality of data coverage, more pavement information about structures, materials, performance deserve future supplement for constructing the KG-PAVEMENT; (2) to realize the intelligence of human-computer interaction, the artificial intelligence question answering system based on the KG-PAVEMENT might merit exploration; (3) to realize intelligent design of pavement structure and accurate prediction of pavement performance based on big data, apart from search engine, the knowledge reasoning system based on the KG-PAVEMENT deserve future investigation.

## Data Availability

The data preasented in this study are available on request from the corresponding author.
